# Understanding the Molecular Mechanism of the Rearrangement of Internal Nitronic Ester into Nitronorbornene in Light of the MEDT Study

**DOI:** 10.3390/molecules24030462

**Published:** 2019-01-28

**Authors:** Agnieszka Kącka-Zych

**Affiliations:** Institute of Organic Chemistry and Technology, Cracow University of Technology, 31-155 Kraków, Poland; akacka@chemia.pk.edu.pl

**Keywords:** molecular electron density theory, bonding evolution theory, electron localization function, molecular mechanism, Diels–Alder reaction, nitro compounds

## Abstract

The characterization of the structure of nitronic esters and their rearrangement into nitronorbornene reactions has been analyzed within the Molecular Electron Density Theory (MEDT) using Density Functional Theory (DFT) calculations at the B3LYP/6-31G(d) computational level. Quantum-chemical calculations indicate that this rearrangement takes place according to a one-step mechanism. The sequential bonding changes received from the Bonding Evolution Theory (BET) analysis of the rearrangement of internal nitronic ester to nitronorbornene allowed us to distinguish seven different phases. This fact clearly contradicts the formerly-proposed concerted pericyclic mechanism.

## 1. Introduction

The Diels–Alder reaction (DA) of nitroalkenes to conjugated dienes is a powerful tool for the synthesis of nitrocarboxylic systems, which can be used as precursors for the synthesis of pharmaceuticals and biomaterials [1,2,3]. However, in some cases, the DA processes can compete with the Hetero-Diels–Alder (HDA) reaction, in which case, it is therefore possible that some of the electrons involved in the reaction come from the bond of the nitro group [4,5,6]. According to the literature, the HDA adduct can be recast into a DA product. It is assumed that this rearrangement of the carboxylic skeleton is analogous to the Claisen rearrangement [7,8] and can be interpreted as [3.3]-sigmatropic shift [9]. In the literature, we find many experimental conformances of these processes. Wade and co-workers proved that the rearrangement of O-allyl nitronic esters (nitronates) obtained in the E-2-phenylnitroethylene and 1,3-cyclohexadiene reaction undergo conversion to the DA product, the γ,δ-unsaturated nitro compound, with high efficiency [10,11]. The necessary nitronic ester precursors are readily obtained by HDA cycloaddition reactions of nitroalkenes with appropriate dienes [10,11,12].

The internal nitronic ester rearrangement has been the subject of several theoretical investigations [13,14,15,16,17,18]. There is a view that the rearrangement of nitronic esters progresses according to a six-membered transition state, and this is considered as a “pericyclic” process [19,20]. Domingo generally negated the term “pericyclic process” for the majority of the defined reactions [21]. Therefore, the mechanistic aspects of this rearrangement necessarily require deep re-examination. 

This paper is a continuation of a comprehensive mechanistic study of the reaction mechanism using the Molecular Electron Density Theory (MEDT) [22,23]. This theory, proposed by Domingo in 2016 [24], in which it is established that changes in electron-density, and not molecular orbital interactions, are responsible for reactivity in organic chemistry, uses quantum chemical tools such as the conceptual Density Functional Theory (DFT) [25,26] reactivity indicator and the topological Electron Localization Function (ELF) [27] analysis of the changes in the molecular electron-density along the reaction path, in order to study organic reactions. Thus, MEDT is an insightful reactivity model in the study of organic reactions, has been applied in many recent theoretical studies and has allowed the performance of many processes in a different light [28,29,30,31,32,33,34,35,36,37]. 

**Scheme 1:**
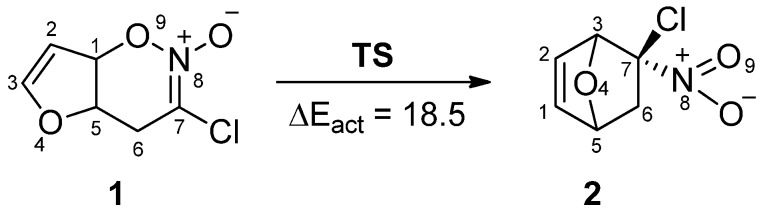
General scheme of the rearrangement of internal nitronic ester **1** into nitronorbornene **2**. B3LYP/6-31G(d) relative energy with respect to **1** is given in kcal·mol^−1^.

In order to understand the rearrangement, an MEDT study [22,23] of the transformation of **1** as a model nitronic ester into nitronorbornene **2** (Scheme 1) was carried out at the B3LYP/6-31G(d) computational level, in which a combination of: (i) ELF topological analysis and a Natural Population Analysis (NPA) of the HDA cycloadduct **1** was performed in order to characterize its electronic structure, (ii) Bonding Evolution Theory (BET) [38] and Noncovalent Interactions (NCIs) study of the transformation of HDA cycloadduct **1** to DA cycloadduct **2**, and (iii) MEDT study of the rearrangement. 

## 2. Results and Discussion

The present MEDT study has been divided into three sections: (i) in Section 2.1. is the ELF and NPA characterization of the structure of internal nitronic ester **1**; (ii) in Section 2.2, the BET and NCIs study of the rearrangement nitronic ester **1** into nitronorbornene **2** are performed; and finally, (iii) in Section 2.3, an MEDT study of the rearrangement is conducted.

### 2.1. ELF and NPA Characterization of the Structure of Internal Nitronic Ester **1**

**Figure 1:**
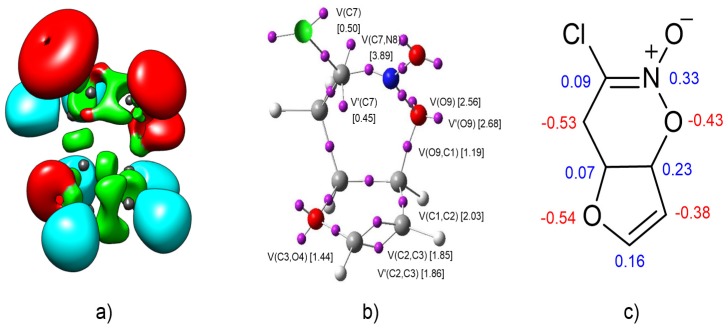
(**a**) ELF localization domains of **1**, represented at an isosurface value of the Electron Localization Function (ELF) = 0.75; (**b**) ELF basin attractor positions, together with the most representative valence basin populations; (**c**) Lewis-like structure of **1** together with the natural atomic charges, obtained through an Natural Population Analysis (NPA). Negative charges are colored in red, and positive charges are colored in blue. ELF valence basin population and natural atomic charges are given in average number of electrons, e.

At the beginning, I decided to investigate the electronic structure of nitronic ester **1**. For this purpose, topological analysis of the ELF and NPA of the **1** was performed. ELF localization domains and their attractors’ positions, together with the most representative valence basin populations, and the proposed Lewis structure with natural atomic changes are shown in Figure 1. 

Topological analysis ELF of **1** showed the presence of four monosynaptic basins, two derived from C7-V(C7) and V’(C7), integrating a total electron density of 0.95e and two of O9-V(O9) and V’(O9) integrating 2.56e and 2.68. We also noted the presence of six disynaptic basins, V(C1, C2), V(C2, C3), V’(C2, C3), V(C3, O4), V(C7, N8), and V(O9, C1) integrating 2.03e, 1.85e, 1.86e, 1.44e, 3.89e, and 1.19e, respectively (Figure 1). In accordance with ELF analysis, monosynaptic basins are associated with non-bonding regions; in turn, disynaptic basins are related to bonding regions [39]. Therefore, within the Lewis bonding model [40], the monosynaptic basins V(O9), V’(O9), V(C7), and V’(C7) can be associated with O9 and C7 lone pairs, the V(C1, C2); V(C3, O4), V(C7, N8), and V(O9, C1) disynaptic basins are related to C-C, C-O, and C-N single bonds; and the V(C2, C3) and V’(C2, C3) disynaptic basins are connected with the double bond between atoms C2 and C3. 

The natural atomic charges, obtained through the NPA [38,41], are given in Figure 1. NPA of **1** indicated that, while the O4 and O9 oxygen and C6 carbon atoms gather the highest negative charges, −0.54e, −0.43e, −0.53e, respectively, the C2 carbon is almost half as negative charged, −0.37e. On the other hand, while the nitrogen N8 presents a relatively high positive charge, 0.33e, the C1 carbon is positively charged by 0.23e, and insignificant positive charges are observed in carbon atoms C5 and C7, 0.07e and 0.09e, respectively. Thus, while the O4, O9, C6, and C2 atoms gather negative charges, the N8, C1, C5, and C7 atoms are positively charged. Considering the Lewis-like structure of **1**, neither the O4 and O9 oxygens, nor the C2 or C6 carbons would ever somehow gather negative charges. Note that the charge distribution obtained through the NPA analysis is not the consequence of the resonance Lewis structure, but rather the consequence of asymmetric electron density delocalization within a molecule resulting from the presence of different nuclei in the molecule.

### 2.2. BET and NCIs Study of the Transformation Nitronic Ester **1** into Nitronorbornene **2**

**Figure 2:**
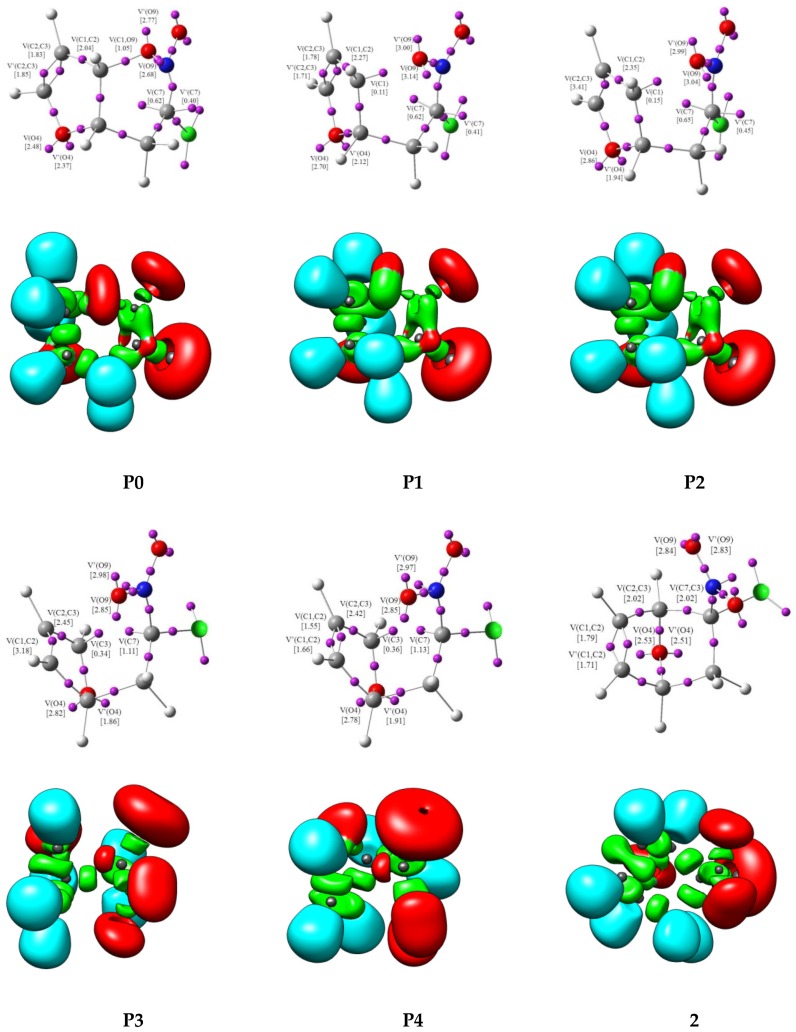
ELF localization domains, represented at isosurface values of ELF = 0.75, together with their attractor positions for the points of IRC defining Phases I–VII along the rearrangement of nitronic ester **1** into nitronorbornene **2**.

**Figure 3:**
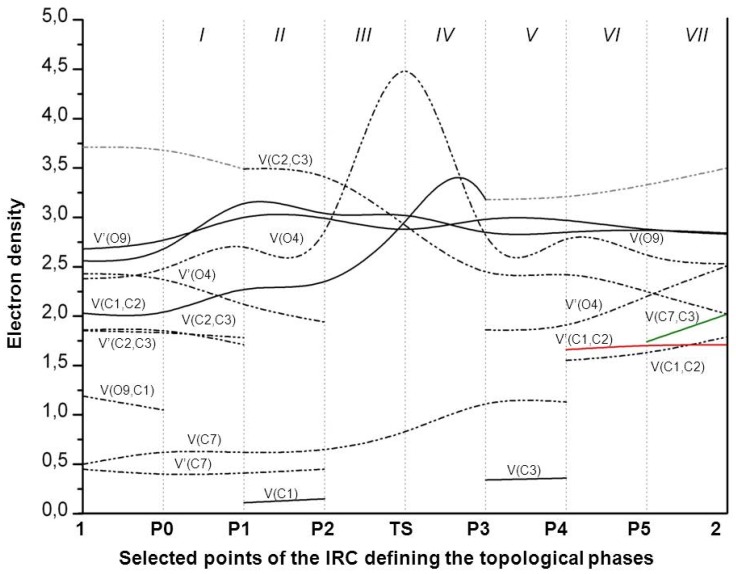
Graphical representation of the basin population changes during the rearrangement of nitronic ester **1** into nitronorbornene **2**. Point dotted curves in grey represent the sum of disynaptic basins describing a bond region or monosynaptic basins describing lone pairs.

The geometries of the transition state (**TS**) and stationary points and Cartesian coordinates from the B3LYP/6-31G(d) calculations for the structures of rearrangement are given in the Supplementary Materials (Tables S4 and S5). The BET study of the transformation of the nitronic ester **1** into 5-chloro-5-nitro-7-oxo-bicyclo [2.2.1]-hept-2-ene **2** indicates that this reaction is topologically characterized by seven different phases. The population of the most significant valence basins of the selected points of the intrinsic reaction coordinate (IRC), **Pi**, defining the different topological phases, is included in Table 1. The attractor positions of the ELF for the relevant points along the IRC are shown in Figure 2. The basin-population changes along the reaction path are graphically represented in Figure 3.

Phase I, 1.53 Å ≤ d(O9-C1) < 1.84 Å and 3.23 Å ≥ d(C7-C3) > 3.19 Å, begins at **P0**, which is the discontinuous point of the IRC from TS towards the isolated nitronic ester **1**. The ELF topological analysis of **P0** divulges slight changes in ELF valence basin electron populations of **1** (see Table 1 and Figure 2). The population of V(O9, C1), V(C2,C3), and V’(C2,C3) disynaptic basins progressively decreases, in contrast with that of the V(C1,C2), which incrementally increases. At this point, in addition, the population of V(O9, C1) reaches the minimum integrating 1.05e. The global electron density transfer (GEDT) value is 0.31[e].

At **P1**, Phase II begins, 1.84 Å ≤ d(O9-C1) < 1.93 Å and 3.19 Å ≥ d(C7-C3) > 3.17 Å, which is described by a cusp C^†^ catastrophe. At this point, the first significant changes along the reaction patch take place; the V(O9, C1) disynaptic basin disappears; and a new V(C1) monosynaptic basin is created integrating 0.11e. We also observed, that the population of V’(C2, C3) reaches the minimum integrating 1.71e. We notice at this point that the GEDT value increases to 0.32[e]. With the changes taking place along this phase, one disynaptic basin V(O9, C1) disappears, with a demand energy cost of 3.6 kcal·mol^−1^ (see Table 1).

Phase III, 1.93 Å ≤ d(O9-C1) < 2.64 Å and 3.17 Å ≥ d(C7-C3) > 2.75 Å, starts at **P2**. This point is characterized by a cusp C^†^ catastrophe. In this phase, we observe the next important topological change along the IRC. While the two V(C2, C3) and V’(C2, C3) disynaptic basins have merged into a new V(C2,C3) disynaptic basin integrating 3.41e, the GEDT is increased to 0.32[e].

At Phase IV, 2.64 Å ≤ d(O9-C1) < 2.93 Å and 2.75 Å ≥ d(C7-C3) > 2.31 Å, which begins at **TS**, the next significant topological change along the reaction path takes place. At this phase, established by a fold F catastrophe, the V(C1) monosynaptic basin disappears, and the V(O4) and V’(O4) monosynaptic basins merge into a new V(O4) monosynaptic basins integrating 4.48e. The transition state **TS** of this rearrangement is found in this phase, d(O9-C1) = 2.641Å and d(C7-C3) = 2.745Å (Table 1, Figure 3). The GEDT value reaches the maximum value and is equal to 0.34[e] (Table 1).

Along Phases III–IV, V’(C2, C3), the disynaptic basin disappears, and two monosynaptic basins merge into a new V(O4) monosynaptic basin. These changes are related to a high energy cost of 13.7 kcal·mol^−1^ (Table 1).

At **P3** begins Phase V, 2.93 Å ≤ d(O9-C1) < 2.78 Å and 2.31 Å ≥ d(C7-C3) > 2.27 Å. This point is characterized by fold F catastrophe, in which the V(O4) monosynaptic basin divides again into two V(O4) and V’(O4) monosynaptic basins. In this phase, we observed the decrease the GEDT value to 0.24[e].

Phase VI, 2.78 Å ≤ d(O9-C1) < 2.81 Å and 2.27 Å ≥ d(C7-C3) > 2.05 Å, characterized by a cusp C^†^ catastrophe, starts at **P4**. At this point, the next most relevant topological change along the reaction patch occurs; the V(C1, C2) disynaptic basin reaches 3.18e and splits into two new V(C1,C2) and V’(C1,C2) disynaptic basins integrating 1.55e and 1.66, respectively. In this phase, we observed the formation of a double bond between atoms C1 and C2. We notice at this point a decrease in the value of GEDT to 0.17[e].

Along Phases V–VI, we observed the creation of the V’(C1, C2) disynaptic basin, and the energy of this change was 6.8 kcal·mol^−1^ (Table 1).

Finally, the last Phase VII, 2.81 Å ≤ d(O9-C1) < 3.70 Å and 2.05 Å ≥ d(C7-C3) > 1.57 Å, is located between points **P5** and **2**. Here, characterized by cusp C^†^ catastrophe, a new V(C7, C3) disynaptic basin integrating 1.74e is created from the two V(3) and V(C7) monosynaptic basins (Table 1). This change can be related to the formation of the C7-C3 bond in nitronorbornene **2**. The GEDT value is 0.12[e].

**Figure 4:**
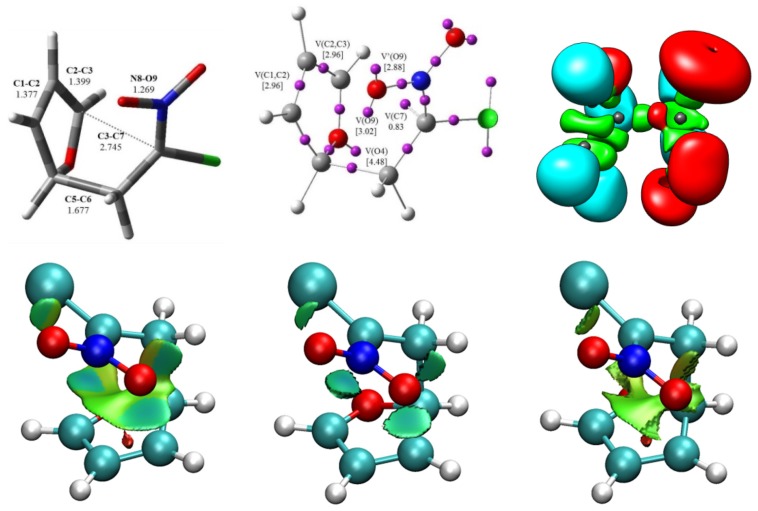
ELF localization domains, represented at isosurface values of ELF = 0.75, together with their attractor positions and analysis of Noncovalent Interactions (NCIs) for the transition state **TS** of the rearrangement of nitronic ester **1** into nitronorbornene **2**.

The geometries of TS with ELF localization domains and attractor positions in this one-step rearrangement process of nitronic ester **1** into the final product are given in Figure 4. It turned out that TS has a six-membered structure (Figure 4). In this transition state, the O9-C1 bond is broken, and a new σ-bond between atoms C3-C7 is formed. **TS** have a polar character, which is confirmed by the GEDT value and dipole moment, which is equal 6.08 D (Table 1).

Finally, an analysis of the NCIs at **TS** was performed. As can be seen in Figure 4, depending on their electrostatic nature, NCIs can be either attractive or repulsive interactions. In **TS** structure of the rearrangement of nitronic ester **1** into nitronorbornene **2**, the weak interactions are mainly repulsive, but the attractive ones may be stronger quantitatively. The weak attractive interactions are between the O-C, C-C, and O-H atoms.

### 2.3. MEDT Study of the Rearrangement of Internal Nitronic Ester **1** into Nitronorbornene **2**

In this section, the MEDT study of the rearrangement nitronic ester **1** into nitronorbornene **2** is presented, and the bonding changes arising from the BET study and their associated energy changes along the shift of **1** are summarized and described in a chemical fashion.

It should be mentioned that DFT study using different M06-2X functionals present the same mechanism of the rearrangement of internal nitronic ester **1** into nitronorbornene **2**. However, these calculations suggest slightly lower relative energy. The complete thermodynamic parameters and relative energies for this rearrangement provided in different functionals and different basic sets are provided in the Supplementary Materials (Tables S1–S3). Thereafter, I also analyze the influence of solvent polarity on the reaction kinetics of this rearrangement of **1** into **2**. In the presence of polar reaction medium, the relative energy is slightly lower (Supplementary Materials, Tables S3 and S4). Thus, regardless of the polarity of the medium, the reaction proceeds as a one-step mechanism.

The sequential bonding changes received from the BET analysis of the rearrangement of nitronic ester **1** are shown in Table 2, together with a simplified representation of the molecular mechanism by ELF-based Lewis-like structures. From the MEDT analysis of this rearrangement, some appealing conclusions can be drawn: (i) the molecular mechanism of the rearrangement of nitronic ester **1** into final product **2** can be topologically characterized by seven different phases, which have been grouped into four groups, A–D, and linked to significant chemical events (see Table 2); according to this fact, we can evidently exclude the pericyclic mechanism [42]; (ii) Group A, containing Phase I and Phase II, is associated with the rupture of the O9-C1 bond of nitronic ester **1**; (iii) Group B comprises Phases III and IV, in which we observed the breaking of the double bond between atoms C2-C3; in this group, we find the transition state **TS** of this rearrangement; the activation energy with this reaction, 18.5 kcal·mol^−1^, can mainly be connected with the rupture of the O9-C1 and C2-C3 bonds of nitronic ester **1**; (iv) Group C containing Phase V is mainly associated with the formation of the C1-C2 double bond; (v) the last Group D contains Phases VI and VII in which we observed the formation of a σ-bond between atoms C3 and C7 in nitronorbornene molecule **2**.

## 3. Computational Methods

Calculations were performed using the Prometheus computer cluster at the CYFRONET regional computer center in Cracow. All calculations were carried out with the GAUSSIAN 16 package [43]. DFT calculations were performed using the B3LYP [44,45] and M06-2X [46] functionals together with the 6-31G(d), 6-311+G(d), and 6-31G(d,p) basis sets [47]. The stationary points were characterized by frequency calculations in order to verify the number of imaginary frequencies (zero for local minima and one for TSs). The IRC [48] paths, computed using the second order González–Schlegel integration method [41,49], were traced in order to obtain the energy profiles connecting TS to the two associated minima of the proposed mechanism. The solvent effects of diethyl ether and nitromethane in the optimizations were taken into account using the Polarizable Continuum Model (PCM) as developed by Tomasi’s group [50,51,52] in the framework of the Self-Consistent Reaction Field (SCRF) [53,54]. Values of enthalpies, entropies, and free energies in all calculations were calculated with the standard statistical thermodynamics at 25 °C and 1 atm [47]. Noncovalent Interactions (NCIs) were computed using the methodology previously described [55,56].

The GEDT [21] was computed by the sum of the natural atomic charges (q), obtained by a Natural Population Analysis (NPA) [57,58], of the atoms belonging to each framework (f) at the **TSs**; i.e., GEDT (f) = ∑qεf q. The sign indicates the direction of the electron density flux in such a manner that positive values mean a flux from the considered framework to the other one.

The topological analyses of the Electron Localization Function (ELF) were performed with the TopMod [38] program using the corresponding B3LYP/6-31G(d) monodeterminantal wavefunctions. ELF calculations were computed over a grid spacing of 0.1 a.u. for each structure, and ELF localization domains were obtained for an ELF value of 0.75. For the Bonding Evolution Theory (BET) [59] studies, the corresponding reaction paths were followed by performing the topological analysis of the ELF for at least 167 nuclear configurations along the IRC paths.

## 4. Conclusions

The characterization of the structure of nitronic ester **1** and its rearrangement into nitronorbornene **2** has been studied within MEDT using DFT calculations at the B3LYP/6-31G(d) computational level. This rearrangement takes place according to a one-step mechanism. According to the BET analysis, the reaction begins by the asynchronous rupture of the O9-C1 bond. In turn, the synchronous rupture of the C2-C3 double bond comes later. These changes are responsible for an energy cost 18.5 kcal·mol^−1^. Then, we observed the formation of the double bond between atoms C1 and C2. The formation of the bond between atoms C7-C3, in molecule **2**, takes place in the last step of the rearrangement. The BET analysis allowed the distinction between the seven different phases in the process of the rearrangement of **1** into **2**, which clearly excludes the pericyclic mechanism, and this rearrangement can be considered as a non-concerted one-step pseudocyclic reaction [30].

## Figures and Tables

**Table 1:** ELF valence basin populations, distances of the breaking and forming bonds, and relative ^a^ electronic energies of the IRC points, **P0**–**P5**, defining the eight phases characterizing the rearrangement of the nitronic ester **1** to 5-chloro-5-nitro-7-oxo-bicyclo[2.2.1]-hept-2-ene **2**. The stationary points **1**, **TS**, and **2** are also included. Distances are given in angstroms, Å, electron populations in average number of electrons, e, relative energies in kcal·mol^−1^, and GEDT values in average number of electrons, [e].

Points	1	P0		P1			P2			TS			P3			P4			P5			2	
*Catastrophes*				*C^†^*			*C^†^*			*F*			*F*			*C^†^*			*C^†^*				
*Phases*			*I*			*II*			*III*			*IV*			*V*			*VI*			*VII*		
d(O9-C1)	1.459	1.533		1.844			1.929			2.641			2.930			2.776			2.814			3.700	
d(C7-C3)	3.472	3.276		3.189			3.170			2.745			2.305			2.266			2.049			1.573	
ΔE ^a^	−4.8	0.0		3.6			9.4			13.7			10.7			6.8			2.1			1.0	
GEDT	0.31	0.31		0.32			0.32			0.34			0.24			0.17			0.14			0.12	
V(C1, C2)	2.03	2.04		2.27			2.35			2.96			3.18			1.55			1.63			1.79	
V(C2, C3)	1.85	1.83		1.78			3.41			2.92			2.45			2.42			2.25			2.02	
V(O4)	2.38	2.48		2.70			2.86			4.48			2.82			2.78			2.62			2.53	
V’(O4)	2.43	2.37		2.12			1.94						1.86			1.91			2.20			2.51	
V(O9)	2.56	2.68		3.14			3.04			3.02			2.85			2.85			2.87			2.84	
V’(O9)	2.68	2.77		3.00			2.99			2.88			2.98			2.97			2.88			2.83	
V(O9, C1)	1.19	1.05																					
V(C7)	0.50	0.62		0.62			0.65			0.83			1.11			1.13							
V’(C2, C3)	1.86	1.85		1.71																			
V’(C7)	0.45	0.40		0.41			0.45																
V’(C1, C2)																1.66			1.70			1.71	
V(C7, C3)																			1.74			2.02	
V(C3)													0.34			0.36							
V(C1)				0.11			0.15																

^a^ Relative to the first point of the IRC, **P0**.

**Table 2:**
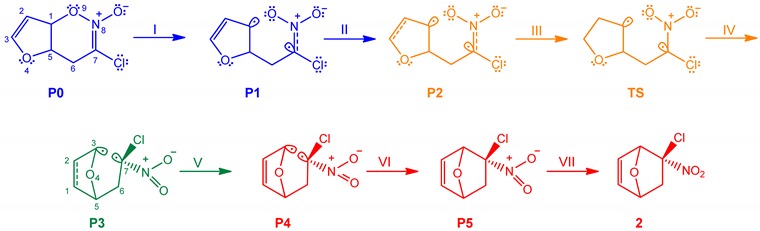
Sequential bonding changes along the rearrangement of the **1** into **2**, showing the equivalence between the topological characterization of the different phases and the chemical processes occurring along them. Distances are given in angstroms, Å, GEDT values in the average number of electrons, [e], and relative energies in kcal·mol^−1^.

Group	Phases	d_1_(O9-C1) d_2_(C7-C3)	GEDT	∆E	Topological Characterization	Chemical Process
A	I–II	1.53 ≤ d_1_ < 1.93 3.28 ≥ d_2_ > 3.17	0.32	3.6	Disappearance of the V(O9, C1) disynaptic basin	Rupture of the O9-C1 bond
B	III–IV	1.93 ≤ d_1_ < 2.93 3.17 ≥ d_2_ > 2.31	0.34	13.7	Disappearance of the V’(C2, C3) disynaptic basin, V(C1) and V’(C7) monosynaptic basins, and joining of the two V(O4) and V’(O4) monosynaptic basins into the V(O4) monosynaptic basin	Rupture of the C2-C3 double bond
C	V	2.93 ≤ d_1_ < 2.78 2.31 ≥ d_2_ > 2.27	0.17	6.8	Formation of the V(C3) monosynaptic basin and the split of the V(C1, C2) disynaptic basin into two V(C1,C2) and V’(C1, C2) disynaptic basins and the V(O4) monosynaptic basin into two V(O4) and V’(O4) monosynaptic basins	Formation of the C1-C2 double bond
D	VI–VII	2.78 ≤ d_1_ < 3.70 2.27 ≥ d_2_ > 1.57	0.12	1.0	Disappearance of V(C7) and V(C3) and formation of the V(C7-C3) disynaptic basin	Formation of the C7-C3 bond

## Supplementary Materials

Table S1: B3LYP thermodynamic parameters and relative energies for the rearrangement of internal nitronic ester 1 to nitronorbornene 2; Table S2: M06-2X thermodynamic parameters and relative energies for the rearrangement of internal nitronic ester 1 to nitronorbornene 2; Table S3: B3LYP/6-31G(d) thermodynamic parameters and relative energies for the rearrangement of internal nitronic ester 1 to nitronorbornene 2 in simulated presence of polar reaction medium; Table S4: B3LYP/6-31G(d) key parameters for the stationary points involved in the rearrangement of 1 into 2 in different reaction medium; Table S5: Cartesian coordinates from the B3LYP/6-31G(d) calculations for the structures of rearrangement of internal nitronic ester 1 to nitronorbornene 2.
