# Supplementary material for: Understanding the Molecular Mechanism of the Rearrangement of Internal Nitronic Ester into Nitronorbornene in Light of the MEDT Study
Source: Molecules. 2019 Jan 28;24(3):462. doi: 10.3390/molecules24030462 (PMC6384843; doi:10.3390/molecules24030462)
Supplement: Supplementary file 1 [file molecules-24-00462-s001.pdf]

Supplementary Material

Understanding the molecular mechanism rearrangement of internal nitronic ester into nitronorbornene in the light of MEDT study

Agnieszka Kącka-Zych

Institute of Organic Chemistry and Technology, Cracow University of Technology; ul. Warszawska 24, 31-155 Cracow, Poland

Email: akacka@chemia.pk.edu.pl

Index

S2 **Table S1.** B3LYP thermodynamic parameters and relative energies for the rearrangement of internal nitronic ester **1** to nitronorbornene **2** (T=298 K; ΔE, ΔH, ΔG in kcal·mol^{-1}, ΔS in cal·mol^{-1}·K^{-1}).

S3 **Table S2.** M06-2X thermodynamic parameters and relative energies for the rearrangement of internal nitronic ester **1** to nitronorbornene **2** (T=298 K; ΔE, ΔH, ΔG in kcal·mol^{-1}, ΔS in cal·mol^{-1}·K^{-1}).

S4 **Table S3.** B3LYP/6-31G(d) thermodynamic parameters and relative energies for the rearrangement of internal nitronic ester **1** to nitronorbornene **2** in simulated presence of polar reaction medium (T=298 K; ΔE, ΔH, ΔG in kcal·mol^{-1}, ΔS in cal·mol^{-1}·K^{-1}).

S5 **Table S4.** B3LYP/6-31G(d) key parameters for the stationary points involved in the rearrangement of **1** into **2** in different reaction medium.

S6 **Table S5.** Cartesian coordinates from the B3LYP/6-31G(d) calculations for the structures of rearrangement of internal nitronic ester **1** to nitronorbornene **2**.

Table S1. B3LYP thermodynamic parameters and relative energies for the rearrangement of internal nitronic ester **1** to nitronorbornene **2** (T=298 K; ΔE, ΔH, ΔG in kcal·mol^{-1}, ΔS in cal·mol^{-1}·K^{-1}).

Basic set	Transition	ΔE	ΔH	ΔG	ΔS
**6-31G(d)**	**1→ TS**	18.5	17.9	32.5	-48.9
	**1→2**	-3.8	-4.4	10.4	-49.6
**6-311+G(d)**	**1→ TS**	20.1	19.5	34.3	-49.7
	**1→2**	-1.1	-1.7	13.4	-50.4
**6-31G(d,p)**	**1→ TS**	18.9	18.4	32.9	-48.8
	**1→2**	-3.2	-3.8	11.0	-49.6

Table S2. M06-2X thermodynamic parameters and relative energies for the rearrangement of internal nitronic ester **1** to nitronorbornene **2** (T=298 K; ΔE, ΔH, ΔG in kcal·mol^{-1}, ΔS in cal·mol^{-1}·K^{-1}).

Basic set	Transition	ΔE	ΔH	ΔG	ΔS
**6-31G(d)**	**1→ TS**	11.7	11.1	25.3	-47.8
	**1→2**	-16.4	-17.0	-2.2	-49.7
**6-311+G(d)**	**1→ TS**	14.8	14.2	29.2	-50.2
	**1→2**	-15.8	-16.4	-1.4	-50.5
**6-31G(d,p)**	**1→ TS**	15.51	14.9	29.7	-49.5
	**1→2**	-15.9	-16.5	-1.7	-49.7

Table S3. B3LYP/6-31G(d) thermodynamic parameters and relative energies for the rearrangement of internal nitronic ester **1** to nitronorbornene **2** in simulated presence of polar reaction medium (T=298 K; ΔE, ΔH, ΔG in kcal·mol^{-1}, ΔS in cal·mol^{-1}·K^{-1}).

	**Transition**	**ΔE**	**ΔH**	**ΔG**	**ΔS**
**Diethyl ether**	**1→ TS**	15.3	14.7	29.4	-49.4
= 4.24)	**1→2**	-3.2	-3.8	10.8	-49.0
**Nitromethane**	**1→ TS**	13.8	13.2	28.0	-49.5
= 36.56)	**1→2**	-2.9	-3.5	11.0	-49.9

Table S4. B3LYP/6-31G(d) key parameters for the stationary points involved in the rearrangement of **1** into **2** in different reaction medium.

**Reaction medium**	**Structure**	**Interatomic distances [Å]**						$\bar{\nu}$	**Dipole moment μ [D]**
		C1-C2	C2-C3	C7-N8	N8-O9	O9-C1	C7-C3		
**Gas phase**	**1**	1.498	1.337	1.311	1.459	1.459	3.472	77.66	4.30
	**TS**	1.377	1.399	1.396	1.269	2.641	2.745	-117.71	6.08
	**2**	1.336	1.527	1.532	1.227	3.939	1.573	52.51	3.17
**Diethyl ether** = 4.24)	**1**	1.496	1.338	1.309	1.445	1.470	3.481	77.86	5.37
	**TS**	1.368	1.410	1.395	1.264	2.806	2.595	-155.77	7.79
	**2**	1.336	1.526	1.531	1.224	3.971	1.573	41.76	3.74
**Nitromethane** = 36.56)	**1**	1.495	1.338	1.308	1.438	1.475	3.486	76.99	5.92
	**TS**	1.364	1.417	1.396	1.262	2.890	2.509	-182.87	8.45
	**2**	1.336	1.526	1.530	1.228	3.981	1.573	39.52	4.00

Table S5. Cartesian coordinates from the B3LYP/6-31G(d) calculations for the structures of rearrangement of internal nitronic ester **1** to nitronorbornene **2**.

Structure	Cartesian coordinates			
**1**	C	0.20097200	-0.98659800	1.24696300
	C	1.06586900	-0.14177800	0.35862700
	N	0.73391500	1.10057300	0.10541700
	H	0.50867900	-2.03148900	1.17286600
	O	1.25858400	1.96318900	-0.59247300
	O	-0.45198800	1.50964400	0.84948400
	Cl	2.46836200	-0.77468200	-0.42316800
	C	-2.06750400	0.64312300	-0.84355900
	C	-1.60302800	0.65438100	0.58031400
	H	-2.39964700	1.51351400	-1.39103100
	H	-2.32502200	1.05947500	1.29551800
	H	0.30423800	-0.67640300	2.29572100
	C	-1.26760800	-0.84171600	0.83232900
	H	-1.93179100	-1.30394200	1.56841300
	C	-1.94118400	-0.60115000	-1.31502800
	H	-2.15010900	-0.99921900	-2.30081500
	O	-1.48547200	-1.52957200	-0.42483700
**P0**	C	0.20087500	-0.98623000	1.24745300
	C	1.06588900	-0.14168100	0.35901100
	N	0.73388600	1.10062600	0.10549300
	H	0.50871000	-2.03111800	1.17366800
	O	1.25881900	1.96310400	-0.59267200
	O	-0.45201500	1.50988600	0.84926900
	Cl	2.46801700	-0.77489900	-0.42326300
	C	-2.06716600	0.64297000	-0.84392000
	C	-1.60309900	0.65461300	0.58009100
	H	-2.39895400	1.51315200	-1.39194800
	H	-2.32524700	1.05977200	1.29509800
	H	0.30402800	-0.67583600	2.29616400
	C	-1.26759000	-0.84158500	0.83241200
	H	-1.93208300	-1.30351200	1.56840300
	C	-1.94132500	-0.60151100	-1.31495000
	H	-2.15001800	-0.99975800	-2.30069500
	O	-1.48498200	-1.52964700	-0.42462900
**P1**	C	0.20069600	-0.98563600	1.24766600
	C	1.06516700	-0.14203400	0.35794600
	N	0.73407500	1.10065800	0.10542600
	H	0.50785500	-2.03076400	1.17477200
	O	1.25949400	1.96311600	-0.59213300
	O	-0.45172300	1.51027100	0.84855600
	Cl	2.46768200	-0.77557700	-0.42294700
	C	-2.06638200	0.64290000	-0.84452600
	C	-1.60313800	0.65492000	0.57956700
	H	-2.39773300	1.51306800	-1.39285800
	H	-2.32550700	1.06079800	1.29397700
	H	0.30419300	-0.67404500	2.29597800
	C	-1.26805500	-0.84087400	0.83291500
	H	-1.93215700	-1.30284000	1.56917900
	C	-1.94036900	-0.60176500	-1.31501400
	H	-2.14857200	-1.00041000	-2.30072900
	O	-1.48586100	-1.52972200	-0.42386400
**P2**	C	0.20103400	-0.98651800	1.24691400
	C	1.06589900	-0.14182000	0.35844700
	N	0.73411000	1.10055800	0.10523700
	H	0.50864800	-2.03142100	1.17282600
	O	1.25901300	1.96322400	-0.59235200
	O	-0.45201700	1.50972500	0.84917700
	Cl	2.46846700	-0.77483500	-0.42313900
	C	-2.06784800	0.64311800	-0.84339800

	C	-1.60305000	0.65443300	0.58033800
	H	-2.40017100	1.51348500	-1.39080500
	H	-2.32490600	1.05952500	1.29570400
	H	0.30439200	-0.67620100	2.29562100
	C	-1.26761200	-0.84159100	0.83241000
	H	-1.93175500	-1.30381000	1.56850600
	C	-1.94157000	-0.60116300	-1.31484500
	H	-2.15075300	-0.99920300	-2.30059700
	O	-1.48565600	-1.52955500	-0.42479300
**TS**	C	-0.18003800	-0.41935200	1.45161100
	C	0.79184700	-0.14802800	0.41116300
	N	1.04149800	1.18223200	0.06853900
	H	-0.02819100	-1.39992900	1.90349000
	O	1.94570500	1.49849700	-0.71640200
	O	0.23204300	2.01519800	0.57899700
	Cl	1.92087500	-1.35507900	-0.12888700
	C	-1.71562600	0.64079400	-1.15550900
	C	-2.06706100	0.78170700	0.16817500
	H	-1.67063900	1.40767800	-1.91590500
	H	-2.40399200	1.67561500	0.67144300
	H	-0.22136400	0.36688500	2.20543000
	C	-1.73464300	-0.48491700	0.82555100
	H	-2.39080000	-0.88372400	1.59692300
	C	-1.28544300	-0.68206500	-1.30225800
	H	-0.90303900	-1.20825700	-2.16685400
	O	-1.57544200	-1.42949300	-0.23454700
**P3**	C	-0.48911600	-0.46893600	1.34917100
	C	0.37486200	-0.24426100	0.08698600
	N	1.20485200	1.04251800	0.11639900
	H	-0.24700800	-1.42864200	1.80735700
	O	1.46821500	1.53145800	-0.97741100
	O	1.55869700	1.47897600	1.20270600
	Cl	1.57471000	-1.56113500	-0.18959300
	C	-1.48151000	1.10373200	-0.86630500
	C	-2.23238500	0.90868700	0.22115600
	H	-1.34194900	2.00169800	-1.45352700
	H	-2.86915200	1.61524700	0.74030000
	H	-0.34792100	0.32362300	2.08431200
	C	-1.90855600	-0.50447500	0.69414500
	H	-2.66966200	-1.04548000	1.25535400
	C	-0.70858600	-0.19963400	-1.05276200
	H	-0.33731600	-0.45683900	-2.04129200
	O	-1.61682300	-1.17776200	-0.54761500
**P4**	C	-0.48902700	-0.46907400	1.34910400
	C	0.37476200	-0.24422300	0.08683900
	N	1.20503500	1.04240500	0.11646500
	H	-0.24691200	-1.42886500	1.80711400
	O	1.46790800	1.53184900	-0.97725100
	O	1.55969900	1.47815600	1.20277700
	Cl	1.57455900	-1.56108600	-0.18997600
	C	-1.48184800	1.10396100	-0.86598300
	C	-2.23258800	0.90867000	0.22150100
	H	-1.34241000	2.00199700	-1.45312800
	H	-2.86949700	1.61501600	0.74076300
	H	-0.34759100	0.32332300	2.08440200
	C	-1.90853100	-0.50453800	0.69424800
	H	-2.66956200	-1.04568000	1.25542700
	C	-0.70872800	-0.19928000	-1.05268800
	H	-0.33755300	-0.45621600	-2.04132800
	O	-1.61678900	-1.17763700	-0.54765500
**P5**	C	-0.48912400	-0.46927500	1.34902400
	C	0.37491000	-0.24439300	0.08686200
	N	1.20474500	1.04256900	0.11650000
	H	-0.24709700	-1.42918300	1.80686500

	O	1.46815800	1.53163700	-0.97729000
	O	1.55841700	1.47899400	1.20282900
	Cl	1.57505500	-1.56085800	-0.18964300
	C	-1.48184200	1.10370400	-0.86617300
	C	-2.23254100	0.90849800	0.22135600
	H	-1.34245900	2.00168500	-1.45340700
	H	-2.86939000	1.61488100	0.74064200
	H	-0.34767500	0.32295700	2.08447900
	C	-1.90853700	-0.50478600	0.69407600
	H	-2.66964000	-1.04587600	1.25522400
	C	-0.70854200	-0.19946500	-1.05279600
	H	-0.33742300	-0.45643500	-2.04145000
	O	-1.61675100	-1.17777300	-0.54779200
**2**	C	-0.48898000	-0.46935400	1.34899100
	C	0.37487500	-0.24430100	0.08677600
	N	1.20471800	1.04255800	0.11652800
	H	-0.24686100	-1.42928600	1.80673900
	O	1.46770900	1.53198200	-0.97725200
	O	1.55885200	1.47860900	1.20284600
	Cl	1.57511100	-1.56077100	-0.18985800
	C	-1.48199700	1.10375700	-0.86595000
	C	-2.23266400	0.90838700	0.22155900
	H	-1.34267100	2.00181800	-1.45307300
	H	-2.86948600	1.61469000	0.74098700
	H	-0.34749600	0.32281400	2.08451200
	C	-1.90843800	-0.50493600	0.69411100
	H	-2.66945600	-1.04614500	1.25524600
	C	-0.70864900	-0.19934900	-1.05274800
	H	-0.33764700	-0.45623300	-2.04146400
	O	-1.61670900	-1.17780200	-0.54778000
